# Docetaxel facilitates lymphatic-tumor crosstalk to promote lymphangiogenesis and cancer progression

**DOI:** 10.1186/s12885-018-4619-8

**Published:** 2018-07-06

**Authors:** Alexandra R. Harris, Matthew J. Perez, Jennifer M. Munson

**Affiliations:** 1 Department of Biomedical Engineering, University of Virginia, Charlottesville, VA 22908 USA; 2 Department of Pathology, University of Virginia School of Medicine, Charlottesville, VA 22908 USA; 3 Department of Biomedical Engineering & Mechanics, Virginia Tech-Wake Forest School of Biomedical Engineering and Sciences, Virginia Polytechnic Institute & State University, Blacksburg, VA 24061 USA

**Keywords:** Triple-negative breast cancer, Tumor-associated lymphatics, Tumor microenvironment, Taxane chemotherapy, Docetaxel, Lymphangiogenesis, Tissue engineered cell culture models, Cancer cell invasion, VEGFC/VEGFR3

## Abstract

**Background:**

Infiltration into lymphatic vessels is a critical step in breast cancer metastasis. Lymphatics undergo changes that facilitate metastasis as a result of activation of the cells lining lymphatic vessels, lymphatic endothelial cells (LECs). Inhibition of activation by targeting VEGFR3 can reduce invasion toward lymphatics. To best benefit patients, this approach should be coupled with standard of care that slows tumor growth, such as chemotherapy. Little is known about how chemotherapies, like docetaxel, may influence lymphatics and conversely, how lymphatics can alter responses to therapy.

**Methods:**

A novel 3D in vitro co-culture model of the human breast tumor microenvironment was employed to examine the contribution of LECs to tumor invasion and viability with docetaxel and anti-VEGFR3, using three cell lines, MDA-MB-231, HCC38, and HCC1806. In vivo, the 4T1 mouse model of breast carcinoma was used to examine the efficacy of combinatorial therapy with docetaxel and anti-VEGFR3 on lymph node metastasis and tumor growth. Lymphangiogenesis in these mice was analyzed by immunohistochemistry and flow cytometry. Luminex analysis was used to measure expression of lymphangiogenic cytokines.

**Results:**

In vitro, tumor cell invasion significantly increased with docetaxel when LECs were present; this effect was attenuated by inhibition of VEGFR3. LECs reduced docetaxel-induced cell death independent of VEGFR3. In vivo, docetaxel significantly increased breast cancer metastasis to the lymph node. Docetaxel and anti-VEGFR3 combination therapy reduced lymph node and lung metastasis in 4T1 and synergized to reduce tumor growth. Docetaxel induced VEGFR3-dependent vessel enlargement, lymphangiogenesis, and expansion of the LEC population in the peritumoral microenvironment, but not tumor-free stroma. Docetaxel caused an upregulation in pro-lymphangiogenic factors including VEGFC and TNF-α in the tumor microenvironment in vivo.

**Conclusions:**

Here we present a counter-therapeutic effect of docetaxel chemotherapy that triggers cancer cells to elicit lymphangiogenesis. In turn, lymphatics reduce cancer response to docetaxel by altering the cytokine milieu in breast cancer. These changes lead to an increase in tumor cell invasion and survival under docetaxel treatment, ultimately reducing docetaxel efficacy. These docetaxel-induced effects can be mitigated by anti-VEGFR3 therapy, resulting in a synergism between these treatments that reduces tumor growth and metastasis.

**Electronic supplementary material:**

The online version of this article (10.1186/s12885-018-4619-8) contains supplementary material, which is available to authorized users.

## Background

Triple-negative breast cancer (TNBC) accounts for approximately 15% of all breast carcinomas. Defined by its lack of expression of estrogen receptor, progesterone receptor, and human epidermal growth factor receptor 2, TNBC is associated with poorer prognosis and higher rates of distant recurrence compared to receptor-positive breast cancers [1, 2]. While receptor-positive breast cancers have clinically-defined targeted therapeutic regimens available, no targeted therapies have been clinically approved for the treatment of TNBC [3, 4]. Therefore, surgery and cytotoxic chemotherapy still represent the standard of care for these patients [4]. Many TNBC patients undergo neoadjuvant chemotherapy treatment in an effort to reduce the size of the primary tumor prior to surgical excision and increase the likelihood of breast conservation [5]. Taxane-based chemotherapy in particular, such as docetaxel, is one of the most widely used chemotherapies in the treatment of both early and metastatic breast cancer, both in multi-chemotherapeutic regimens and as a single agent [6, 7]. However, randomized prospective studies have shown that although neoadjuvant chemotherapy regimens including taxane agent paclitaxel increase the pathologic complete response rate, it does not improve overall survival [8]. Despite having higher initial clinical response rates to chemotherapy, the majority of TNBC patients eventually experience recurrence at metastatic sites [4]. The aggressive nature of these tumors makes preventing progression to metastatic disease a priority in therapeutic strategy.

In breast cancer, metastasis is thought to occur preferentially through the lymphatic system and tumor-associated lymphatic vessel involvement has been found to be an important prognostic indicator [1, 9, 10]. Expression of the Vascular Endothelial Growth Factor C (VEGFC), an activating ligand to the VEGF Receptor 3 (VEGFR3) specific to the tumor-associated lymphatic endothelium, is associated with increased lymphatic vessel density, lymphovascular invasion, and overall poorer prognosis in both murine models of breast cancer and patients [1, 9, 11, 12]. VEGFC:VEGFR3 signaling can increase proliferation of lymphatic endothelial cells (LECs) to induce lymphangiogenesis [13]. Triple-negative disease often has significantly higher levels of VEGFC secretion in the tumor microenvironment, as well as increased lymphatic vessel density and lymphovascular invasion, when compared to receptor-positive subtypes [1, 9, 10]. The correlation between heightened lymphatic involvement and the risk of metastatic spread in triple-negative breast cancer suggests it may be an especially suitable candidate for therapeutic inhibition of lymphangiogenesis to reduce distant recurrence.

Inhibition of VEGFR3 signaling has been shown to reduce metastasis of cancer in multiple murine models, including lung, colorectal, and breast cancer [14–19] and anti-VEGFR3 monoclonal antibodies are currently in early phase clinical trials for solid tumors (NCT01288989). Alishekevitz, et al. recently provided evidence suggesting that paclitaxel, a member of the taxane-based chemotherapy family, promotes VEGFR3+ macrophage homing to treated tumor sites and that this may lead to increased Lyve1+ staining in tissues [20]. This combination of therapy resulted in reduced malignancy of breast cancer in mice. Thus, there is evidence that targeting lymphatics with anti-VEGFR3 therapy in combination with the current standard of care (i.e. taxanes) for TNBC patients may lead to better outcomes; however, further investigation is needed to expand our understanding of these therapies with the tumor microenvironment.

Docetaxel is the most commonly used taxane against TNBC, yet its effects on lymphangiogenesis have not been elucidated. Here we employ a quantitative phenotypic characterization of docetaxel-induced changes to lymphatic vessels in vivo, as well as a unique human 3D in vitro co-culture model of the tumor-lymphatic interface, to provide novel insight into docetaxel-induced lymphatic-tumor crosstalk. Further, through multiplex analysis, we identify a host of cytokine changes that contribute not only to docetaxel-induced lymphangiogenesis, but also to VEGFR3-mediated tumor growth. Together, these studies illustrate a counter-therapeutic effect of a commonly used chemotherapy, providing novel insight into how docetaxel influences lymphatics to promote cancer spread and how these activating changes could be mitigated by adjuvant blockade of VEGFR3 signaling.

## Methods

### Cell culture

Human mammary cancer cell lines HCC38 (ATCC, Cat. # CRL-2314) and HCC1806 (ATCC, Cat. # CRL-2335), generously given by the Janes laboratory at University of Virginia, were cultured in Roswell Park Memorial Institute (RPMI, Gibco) medium, and MDA-MB-231 (ATCC, Cat. # HTB-26) were acquired from the ATCC and cultured in Dulbecco’s modified Eagle’s medium (DMEM, Gibco), both supplemented with 10% fetal bovine serum (FBS). Human LECs (HMVEC-dLy, Lonza, Cat. #CC-2810) were cultured in Endothelial Cell Growth Medium (EBM-2 basal media, Lonza) supplemented with recommended growth supplement kit (EGM-2MV BulletKit, Lonza). Human mammary fibroblasts (Sciencell, Cat. # 7630) were cultured in basal Fibroblast Medium (FM, Sciencell) supplemented with Fibroblast Growth Supplement (FGS, Sciencell), Penicillin/Streptomycin (Sciencell), and 20% FBS (Sciencell) on tissue-culture treated flasks coated with Poly-L-Lysine (Sciencell). Mouse mammary carcinoma cell line 4T1-luc-red (generously given by the Cross laboratory at University of Virginia) originated from ATCC (Parental line Cat. # CRL-2539) and was acquired from Perkin-Elmer (Cat. # BW124087V) after lentiviral transduction of Red-FLuc luciferase gene. 4T1 cells were cultured in RPMI medium supplemented with 10% FBS. All cell lines were grown sterilely in a humidified atmosphere of 5% CO2 and 95% oxygen at 37 °C. Cell lines were tested annually for mycoplasma (last test date: 12/2015, negative) and all experiments were completed afterwards.

### 3D in vitro co-culture model [25]

10,000 LECs were seeded on the underside of 8 μm pore size 96-well tissue culture inserts (Corning). After 48 h, 50 μl of a Rat Tail Collagen I (Corning)/basement membrane extract (Trevigen) (0.18 mg/ml Collagen, 0.5 mg/ml BME) containing Cell Tracker dye (ThermoFisher)-labeled human mammary fibroblasts (100,000 HMF/ml) and human breast cancer cells (660,000 TNBC cells/ml) was placed atop the inserts. After gelation, media with or without MAZ51 (1 μM, Enzo Life Sciences) was added to the bottom compartment and flow was applied via a pressure head in the top compartment overnight (~ 1 μm/s; 16-18 h), after which point docetaxel was applied via flow to the top compartment and media with or without MAZ51 was again added to the bottom compartment for 24 h and then flushed from the system with basal media. 48 h after drug application, gels were removed, dissociated using Liberase TM (Roche), and processed for flow cytometry. Inserts were processed for invasion analysis. All experimental conditions were run as triplicate samples in individual inserts.

### Invasion analysis

After gel was removed, tissue culture inserts were washed briefly in phosphate-buffered saline (PBS) and fixed with 4% paraformaldehyde. Inserts were stained with DAPI and visualized by fluorescence microscopy. Cancer cells (DAPI+ Cell Tracker Deep Red+) were counted in five individual fields per well. Percent cancer cell invasion was calculated as previously described [21, 25]. Three technical replicates were averaged for each experimental run to give a single biological replicate value for statistical analysis.

### Flow cytometry

Cells were washed and stained with Live/Dead Fixable NIR dye (Life Technologies). Gels were pooled from three inserts prior to degradation to yield a single value for each experimental run. Cells from in vivo digested tissue were stained with live/dead reactive dye, anti-mouse CD45 PerCP-Cy5.5 (eBioscience), anti-mouse CD31 FITC (eBioscience), and anti-mouse gp38 PE-Cy7 (eBioscience). Flow cytometry samples were processed using the Millipore Guava easyCyte 8HT Flow Cytometer and analyzed using InCyte software.

### Cell viability assay

Conditioned media was generated from cultured human LECs for 24 h. A flask with no cells was ‘cultured’ at 37 °C for the same amount of time in parallel with the same media composition to serve as control. Three cancer cell lines were plated alone at equal densities in a 96-well plate and treated with LEC-conditioned media and 1 μM of docetaxel simultaneously. 24 h later, 10 μL of CCK8 viability solution (Enzo Life Sciences) was administered to each well and allowed to incubate for 1.5 h. Absorbance values were measured at 450 nm.

### Syngeneic orthotopic tumor models

Female 5–6 week old Balb/c mice (Jackson Labs) were used in all animal studies. All animal procedures were conducted in accordance with the University of Virginia Institutional Animal Care and Use Committee (Charlottesville, VA).

#### Tumor growth tracking with treatment

50,000 4T1 cells were suspended in phosphate-buffered saline (PBS) with 3 mg/ml growth-factor reduced matrigel and injected sub-areolar into the left 4^th^ mammary fat pad of female Balb/c mice. Once tumors were palpable (7 days post-inoculation), mice underwent three consecutive days of treatment beginning the following day consisting of anti-VEGFR3 antibody (200 μg, I.P., eBioscience (now ThermoFisher), Control IgG: rat monoclonal IgG2a kappa Isotype Control, Cat.#16–4321-85; Anti-VEGFR3: rat monoclonal IgG2a kappa to mouse VEGFR3 (AFL4): Cat. #16–5988-85) or IgG control on the first day, followed by a single dose of docetaxel in saline/20% ethanol (8 mg/kg I.V., Enzo Life Sciences) or saline/20% ethanol control on the second day, and another treatment with anti-VEGFR3 antibody or IgG control on the third day. Tumor volume was measured by caliper every other day once tumors were palpable and calculated by tumor volume = π (L*W)^2^/6 and imaged using IVIS on the final day following d-luciferin injection. Mice were euthanized by CO_2_ inhalation once largest tumor volume reached pre-determined endpoint of 150 mm^3^. Tumor-bearing mammary fat pads and contralateral naïve fat pads were harvested and processed for either lymphatic analysis or lymph node metastasis by immunohistochemistry (fixation with 4% paraformaldehyde) or homogenized for lymphatic drainage (see Evans Blue Drainage Assay below) or protein analysis (see ELISA and Luminex Multiplex Analysis).

#### Metastasis analysis in 4T1 with treatment

250,000 4T1 cells were suspended in phosphate-buffered saline (PBS) with 3 mg/ml growth-factor reduced matrigel and injected sub-areolar into the left 4^th^ mammary fat pad of female Balb/c mice. Once tumors were palpable (5 days post-inoculation), mice underwent three consecutive days of treatment beginning the following day consisting of anti-VEGFR3 antibody (200 μg, I.P., eBioscience) or IgG control on the first day, followed by a single dose of docetaxel in saline/20% ethanol (8 mg/kg I.V., Enzo Life Sciences) or saline/20% ethanol control on the second day, and another treatment with anti-VEGFR3 antibody or IgG control on the third day. Tumor volume was measured by caliper every other day once tumors were palpable and calculated by tumor volume = π (L*W)^2^/6. Mice were euthanized by CO_2_ inhalation once largest tumor volume reached pre-determined endpoint of 300 mm^3^. Mammary fat pads were harvested and processed for flow cytometry (dissociation as previously described [22]) and total LEC counts quantified (see Flow Cytometry). Lungs were harvested, cryoembedded, sectioned, and stained with hematoxylin and eosin (H&E) to quantify metastases.

### Evans blue drainage assay

25 ul of 20 mg/ml Evans blue dye (Sigma-Aldrich) was delivered directly into the tumor-bearing mammary fat pad of experimentally treated mice via subareolar injection slowly over a two-minute period. Dye was allowed to drain for 2 h after injection, and mice were then euthanized and tumor-draining axillary lymph nodes were harvested, weighed, washed, homogenized in TPER buffer, spun at 10,000 rpm for 5 min to remove debris, and protein harvested as described for ELISA below. Lymph node homogenates were read on a microplate reader for absorbance at 620 nm and compared against a sixteen-point standard curve of Evans blue dye; results are normalized to tissue weight and shown as μg Evans blue per mg axillary lymph node tissue.

### ELISA and Luminex multiplex analysis

Tissue was homogenized with TPER buffer with protease and phosphatase inhibitors. Protein was harvested by centrifugation and quantified by BCA assay (Pierce). Vascular endothelial growth factor C was measured by ELISA (Quantikine kit, R&D Systems) and the plate was analyzed using a microplate reader. Luminex array was performed through the UVa Flow Cytometry Core Facility using a Millipore 44-plex cytokine array. Matlab software was used to generate heat map of log-transformed data.

### Immunohistochemistry

Tumor-bearing mammary fat pads were dissected from mice and post-fixed in 4% PFA for 48 h at 4 °C. Tissues were transferred to 70% ethanol for 24 h, dehydrated, and paraffin-embedded. Tissues were sectioned at 7 μm thickness. Sections were stained with anti-podoplanin antibody (1 μg/ml, R&D Systems) followed by ImmPRESS HRP anti-goat IgG peroxidase/SG peroxidase detection (Vector Labs) and nuclear counter-staining with Methyl Green (Vector Labs) was performed. Slides were scanned at 20X on an Aperio Scanscope and individual podoplanin+ vessels were selected from each mouse from each cohort (*n* = 5/cohort, 20–30 vessels/cohort) and analyzed manually for vessel area and perimeter in ImageJ. All lymphatic (podoplanin+) vessels in mammary fat pad were counted and vessel number was normalized to size of stromal area for each mouse and averaged among cohorts to assess lymphatic vessel density as lymphatic vessel #/mm^2^ stroma. For lymphatic metastasis, sections were stained with anti-RFP antibody (5 μg/ml, Thermo Fisher, RFP Tag Monoclonal Antibody RF5R) and whole node confocal scans were used to quantify percent metastatic area of total node.

### Statistical analysis

All data are presented as mean ± standard error of the mean (SEM). Independent *t* test and two-way ANOVA was used for statistical analysis of unmatched groups, while paired *t* tests were used for matched group comparison. Statistical analyses were run using Graphpad Prism software. Tumor growth curves were analyzed by Multivariate ANOVA (MANOVA) using SPSS software package. *p < 0.05* is considered statistically significant. All assays were performed with a minimum of three biological replicates (*n* ≥ 3). Graphs were generated using Graphpad Prism software.

## Results

### Docetaxel treatment increases cancer cell invasion towards lymphatic endothelial cells

**Fig. 1:**
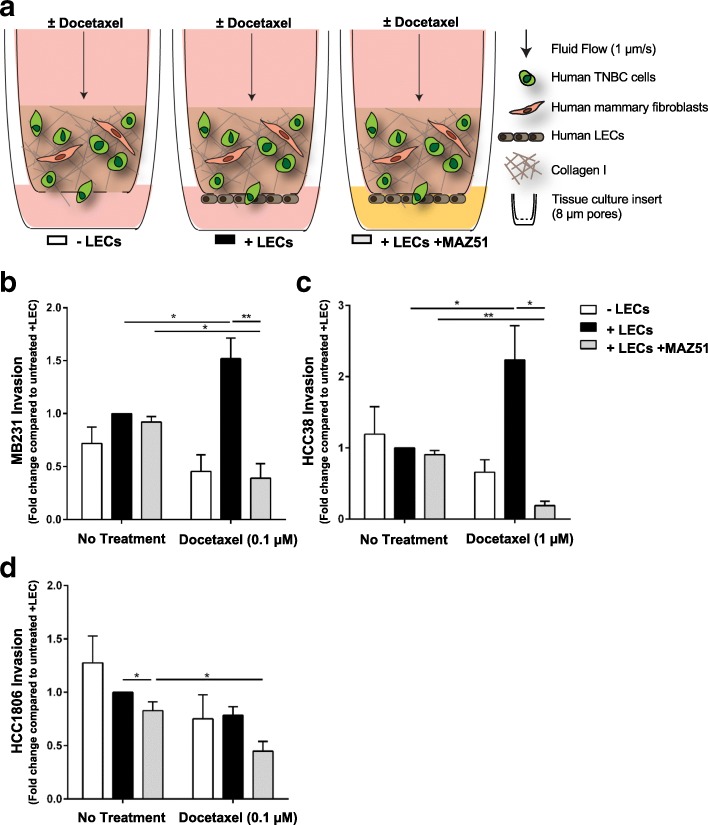
Docetaxel induces invasion of multiple human breast cancer cell lines toward lymphatics in vitro in a VEGFR3-dependent manner. **a** Schematic of the in vitro tissue engineered model of the tumor-lymphatic interface in the human breast cancer microenvironment. Our model contains mammary stromal fibroblasts and TNBC cells in a collagen I matrix. LECs are seeded along the underside of the insert system through which tumor cells transmigrate from a basal to luminal fashion. Physiologically relevant flow (1 μm/s) is applied via a pressure head of media to yield delivery of docetaxel. Schematic depicts experimental groups. **b** Fold change in invasion of MDA-MB-231 tumor cells across the porous membrane in our 3D microenvironment system +/− docetaxel treatment (0.1 μM), +/− MAZ51 (1 μM), and/or in the presence or absence of LECs. **c** Fold change in invasion of HCC38 tumor cells across the porous membrane in our 3D microenvironment system +/− docetaxel treatment (1 μM), +/− MAZ51 (1 μM), and/or in the presence or absence of LECs. **d** Fold change in invasion of HCC1806 tumor cells across the porous membrane in our 3D microenvironment system +/− docetaxel treatment (0.1 μM), +/− MAZ51 (1 μM), and/or in the presence or absence of LECs. Fold change calculated as compared to no docetaxel/with LEC control. *n* ≥ 3 biological replicates. *p < 0.05; **p < 0.01

The tumor microenvironment has been shown to have a powerful influence on cancer growth and metastasis. However, while chemotherapy remains the most commonly employed treatment in the clinical management of breast cancer, studies aiming to understand intercellular interactions between tumor and stroma are rarely performed in the context of therapy. Consequently, very little is known regarding how stromal cell types shown to be important in breast cancer progression, such as LECs, behave under the influence of the standard of care treatment for TNBC patients, cytotoxic chemotherapy. For example, while some in vitro models have shown that LECs can have chemoattractive influences on cancer cells [23, 24], it is unclear whether these phenomena remain true in a chemotherapy-treated microenvironment. We used our previously described human 3D in vitro model of the breast tumor-lymphatic interface [25] to examine the interaction of docetaxel with LECs (Fig. 1a). Given that lymphatic involvement is increased in TNBC and contributes to increased metastasis and poor prognosis in patients, we examined how the presence of LECs contributes to breast cancer cell invasion with and without docetaxel treatment in three different human TNBC cell lines, MDA-MB-231, HCC 38, or HCC 1806 (Fig. 1b-d, Additional file 1: Figure S1). While LECs caused no significant difference in cancer cell invasion in the untreated conditions, we observed significant increases with docetaxel in MDA-MB-231 (Fig. 1b) and HCC38 (Fig. 1c), while HCC1806 remained less LEC-responsive, with only negligible differences (Fig. 1d). Docetaxel alone did not increase invasion of MDA-MB-231 and HCC38 unless LECs were present. HCC1806 did not show increased invasion in the presence of LECs or docetaxel. These data indicate that docetaxel increases invasion of some human breast cancer cell lines toward LECs in a human 3D model of the breast tumor microenvironment.

### VEGFR3-targeted treatment synergizes with docetaxel to reduce cancer cell invasion and metastasis

Since docetaxel did not reduce tumor cell invasion towards lymphatics, but instead induced invasion in some cases, we wished to implement a therapeutic approach to attenuate this lymphatic-dependent effect. Anti-VEGFR3 therapy has shown benefit in reducing metastasis in vivo [14–19] as well as success in pairing with paclitaxel [20]. Given the role of VEGFR3 in the activation and proliferation of tumor-associated LECs, as well as its specificity to lymphatics, we hypothesized that use of a VEGFR3-targeted therapy may reduce invasion of tumor cells towards LECs after docetaxel treatment. We therefore treated the LECs in our in vitro system with MAZ51, a small molecule inhibitor against VEGFR3, while delivering docetaxel as before. We found that while VEGFR3 inhibition using MAZ51 alone did not significantly reduce breast cancer cell invasion, combining it with docetaxel therapy resulted in a significant reduction compared to MAZ51 alone in MDA-MB-231 (Fig. 1b) and HCC38 (Fig. 1c). HCC1806 cells (Fig. 1d) showed an overall reduction in cancer cell invasion in response to docetaxel regardless of VEGFR3 inhibition, further suggesting its LEC-independence.

### VEGFR3 blockade inhibits docetaxel-induced metastatic spread of 4T1 tumor cells

**Fig. 2:**
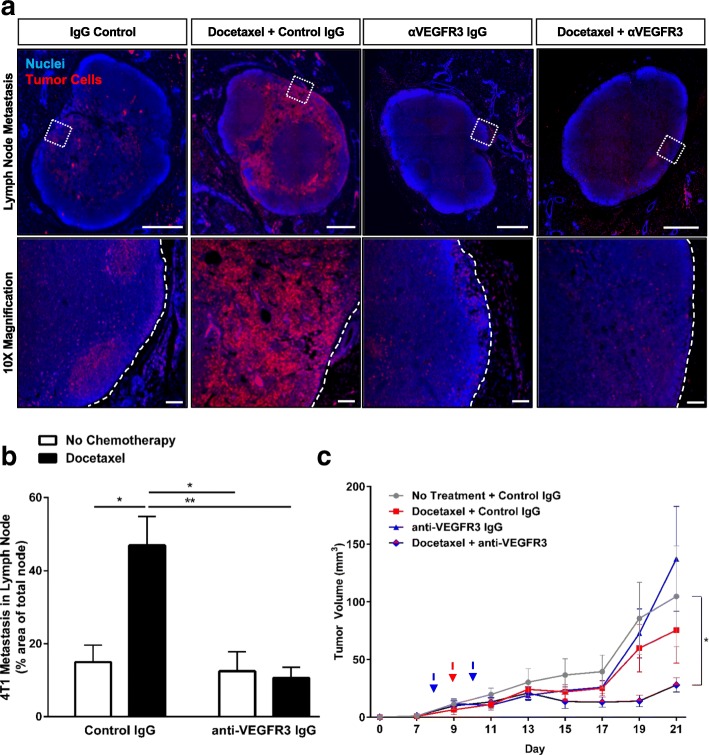
Blockade of VEGFR3 synergizes with docetaxel to reduce tumor growth and docetaxel-enhanced metastasis in 4T1 breast cancer. **a** *Top panel*, Representative images of 4T1 breast tumor cells (red) in the inguinal lymph nodes of mice treated with systemic docetaxel, 8 mg/kg IV (or vehicle control) and/or anti-VEGFR3 antibody, 400 μg total over 2 doses, IP (or control IgG) as detected by histological analysis of RFP-expressing tumor cells. Scale bar = 500 μm. *Bottom panel,* magnified images from boxed regions in top panel. Dotted white lines outline lymph node border. Scale bar = 100 μm. **b** Quantification of lymph node metastasis from whole lymph node scans as percent coverage of RFP+ area in whole lymph node sections. (*n* ≥ 4/group) (**c**) Tumor volume of treated mice (mm^3^) of 4T1 mice treated as described above via caliper measurements. Blue dashed arrow indicates dosing of anti-VEGFR3 antibody or control IgG and red dashed arrow indicates dosing of docetaxel or vehicle control. Curve was analyzed by MANOVA (*n* = 5/group) with *p < 0.05, **p < 0.01

To understand how the docetaxel-induced increase in invasion we observed in our in vitro system translates to metastatic outcomes in vivo, we employed the 4T1 syngeneic orthotopic model of mammary carcinoma. We histologically examined whole inguinal lymph nodes of 4T1 mice treated with a single dose of docetaxel once tumors were palpable. Mice treated with docetaxel exhibited significantly higher numbers of metastatic tumor cells in the lymph nodes, resulting in an over two-fold increase in tumor cell positive area in the node when compared to control IgG mice (Fig. 2a, b).

Given our success using VEGFR3 inhibition to mitigate invasion toward lymphatics in vitro, we next sought to determine if this same therapeutic combination strategy could reduce our observed metastasis in vivo. To this end, we sandwiched our docetaxel treatment between two anti-VEGFR3 treatments and analyzed metastatic outcomes (Fig. 2a, b, Additional file 1: Figure S2). Anti-VEGFR3 therapy with or without docetaxel significantly decreased lymph node metastasis below control levels (Fig. 2a, b). Examination of the lungs of these mice revealed a significant reduction in both the number of metastatic foci per mouse and the number of mice that developed lung metastases in each cohort (Additional file 1: Figure S2A, B). In contrast with its increase of lymph node metastasis, docetaxel treatment did not result in an increase in lung metastasis. Our data illustrate that VEGFR3 inhibition can counteract docetaxel-induced cancer cell invasion and metastasis toward lymphatics.

### Lymphatics decrease docetaxel cytotoxicity

In addition to cancer metastasis, we examined the growth of the primary tumor in response to combination therapy (Fig. 2c) with this same therapeutic regimen through daily caliper measurements and at endpoint using bioluminescent imaging (Additional file 1: Figure S2C). As has been documented with other tumor models and anti-VEGFR3 treatment [14, 17], growth was not affected by anti-VEGFR3 therapy alone. We also did not observe a significant growth decrease with the single dose of docetaxel alone. Interestingly, while single agents alone proved insufficient to reduce tumor growth compared to IgG-treated mice, combination therapy significantly reduced tumor growth in this model. Thus, while lymphatic-targeted anti-VEGFR3 therapy alone shows no effect on tumor growth, it can synergize with docetaxel to potentially enhance chemosensitivity and significantly reduce tumor growth in 4T1 mammary carcinoma.

**Fig. 3:**
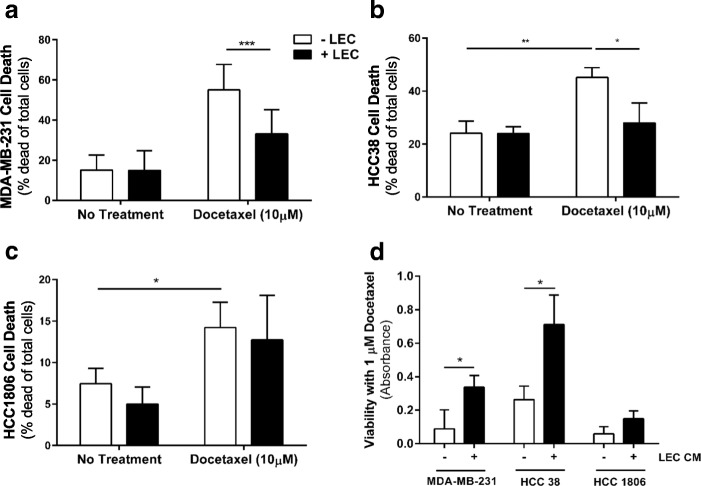
Lymphatic endothelial cells diminish cancer cell response to docetaxel. **a** Cancer cell death (% dead cells of total) of MDA-MB-231 cells in 3D microenvironment system +/− docetaxel treatment (10 μM) +/− LECs as measured by live/dead stain in flow cytometry. **b** Cancer cell death (% dead cells of total) of HCC38 cells in 3D microenvironment system +/- docetaxel treatment (10 μM) +/− LECs. **c** Cancer cell death (% dead cells of total) of HCC1806 cells in 3D microenvironment system +/- docetaxel treatment (10 μM) +/− LECs. **d** LEC-conditioned media (LEC CM) was administered to MDA-MB-231, HCC38, or HCC1806 cells followed by 1 μM docetaxel and viability assessed by CCK8 analysis. Results displayed as measured absorbance. *p < 0.05, **p < 0.01, ***p < 0.001

Since inhibiting lymphatic involvement with anti-VEGFR3 treatment seemed to sensitize 4T1 tumors to docetaxel, we hypothesized that lymphatics may have a direct effect on docetaxel efficacy. We therefore analyzed cancer cell death in our in vitro system with and without LECs after docetaxel treatment (Fig. 3). In untreated conditions, there was no significant difference in cancer cell death when LECs were and were not present across all three TNBC lines. Docetaxel alone increased cancer cell death by two-fold or more across all cell lines. However, in the presence of LECs, MDA-MB-231 (Fig. 3a) and HCC38 (Fig. 3b) cell death was significantly reduced to near untreated levels. This effect was not seen in HCC1806 (Fig. 3c). Dose-response studies in our 3D system show a multi-fold increase in EC50 when LECs are present in both MDA-MB-231 cells (0.3 μM without LECs vs. 81.2 μM with LECs) and HCC38 cells (0.052 μM without LECs vs. 101.2 μM with LECs) (Additional file 1: Table S1). HCC1806 cells were not as sensitive to docetaxel and showed no significant difference in EC50 (513.7 μM without LECs vs. 657.0 μM with LECs). Combination therapy with docetaxel and MAZ51 did not increase cancer cell death in any cell line compared to docetaxel alone (Additional file 1: Figure S3A-C), suggesting that the chemoprotective effect of LECs is VEGFR3-independent.

In order to determine if 1) LECs require tumor education to induce chemoprotection in cancer cells and 2) if docetaxel activates LECs to encourage chemoprotection in tumor cells, we tested drug response by performing a conditioned media study in 2D. LEC-conditioned media was administered to each cancer cell line and cell viability of cancer cells was measured after treatment with docetaxel for 24 h (Fig. 3d). Indeed, LEC-conditioned media-treated cancer cells (MDA-MB-231 and HCC38) showed less death in response to docetaxel when compared to no-LEC media controls. This was true regardless of either any preincubation with tumor-conditioned media or docetaxel treatment. Therefore, we have seen that the presence of LECs alone can decrease response to docetaxel in MDA-MB-231 and HCC38, but not HCC1806.

### Docetaxel induces lymphatic vessel enlargement and lymphangiogenesis in vivo

**Fig. 4:**
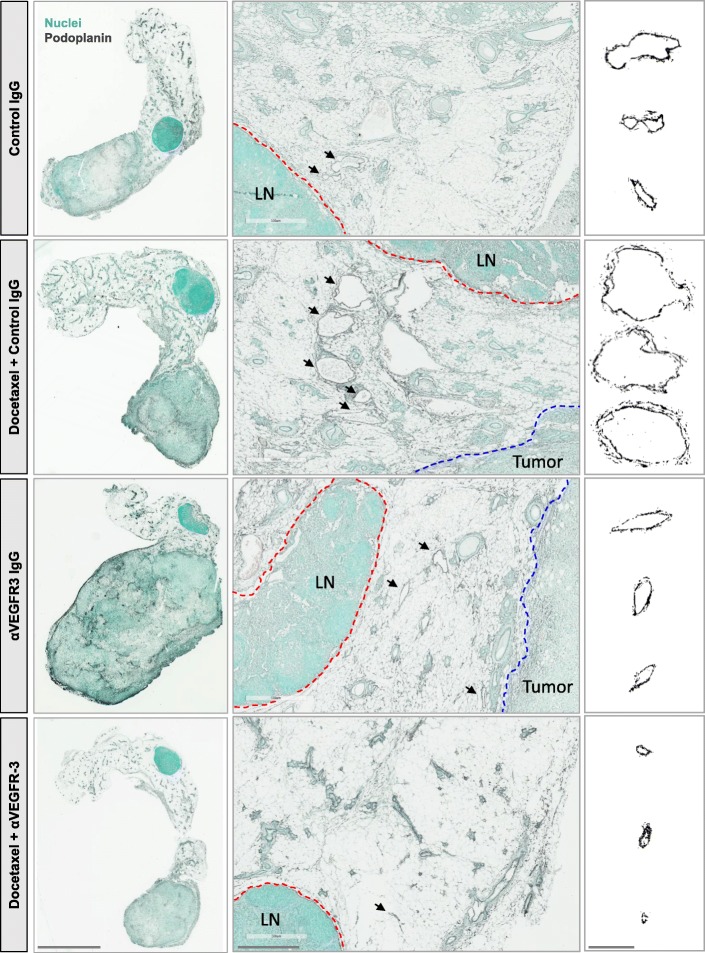
Docetaxel induces VEGFR3-dependent morphological changes of tumor-draining lymphatics in vivo. Representative images of 4T1 tumor-bearing fat pad tissue sections from mice treated as outlined in Fig. 1. Sections were immunostained for lymphatic marker podoplanin (black) and nuclei (green). *Left panel* (scale bar = 4 mm) shows whole tissue sections and *middle panel* (scale bar = 500 μm) shows peritumoral stroma. Red dashed lines show border of inguinal lymph node, blue dashed line shows border of tumor, and black arrows indicate lymphatic vessels. *Right panel* (scale bar = 125 μm) shows representative images of morphology of individual lymphatic vessels

**Fig. 5:**
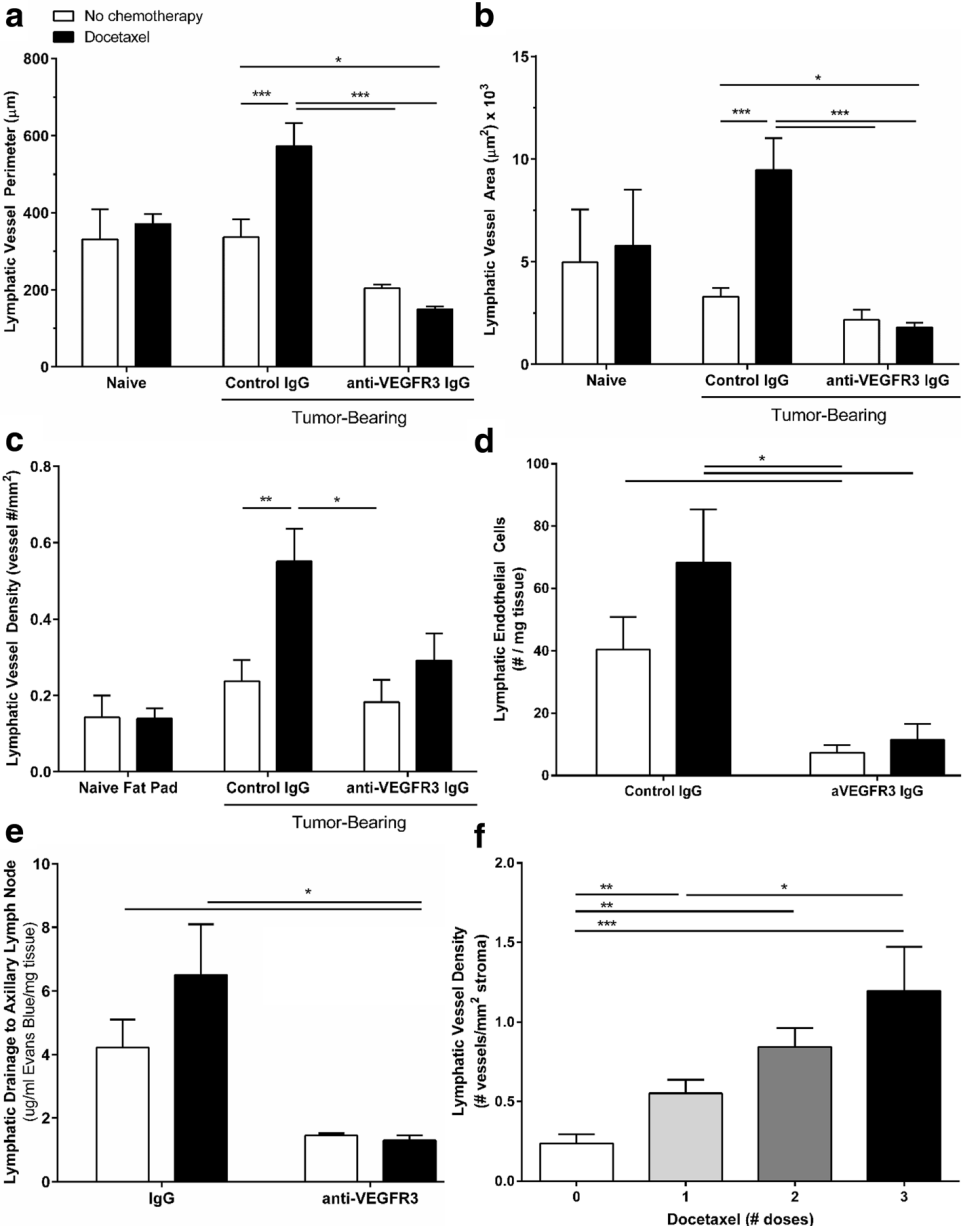
Docetaxel induces enlargement and expansion of tumor-draining lymphatics in vivo that can be attenuated by VEGFR3 blockade. **a** Quantified lymphatic vessel perimeter in both tumor-bearing mammary fat pad of treated 4T1 mice and contralateral naïve (non-tumor bearing) fat pad. **b** Quantified lymphatic vessel area in both tumor-bearing mammary fat pad of treated 4T1 mice and contralateral naïve (non-tumor bearing) fat pad. **c** Quantified lymphatic vessel density of single podoplanin+ lymphatic vessels per stromal area throughout sections. **d** Total lymphatic endothelial cells per weight of fat pad (live CD45^−^CD31^+^gp38^+^) as determined by flow cytometry. **e** Fluid drainage from tumor to axillary lymph node as determined after Evans blue injection into tumor-bearing mammary fat pad 24 h after treatment with docetaxel. (*n* = 5–9 mice per group). **f** Quantified lymphatic vessel density displayed as number of peritumoral lymphatic vessels per mm^2^ of stromal tissue after 0, 1, 2, and 3 doses of docetaxel (8 mg/kg, IV, 3 days apart). *p < 0.05, **p < 0.01, ***p < 0.001

In vivo, anti-VEGFR3 therapies have been shown to reduce lymphangiogenesis, thus reducing the total number of LECs in tissues. We hypothesized that tumor-induced lymphangiogenesis may contribute to reduced therapeutic response with docetaxel in vivo*.* Therefore, we analyzed peritumoral lymphatic vessels in the tumor stroma (Fig. 4). Consistent with findings in breast cancer patients that often show enhanced peritumoral lymphangiogenesis but no intratumoral lymphangiogenesis, intratumoral vessels were rare in these murine tumors and therefore not quantified. Tumor-associated peritumoral lymphatics showed dramatic morphological differences across treatment conditions; lymphatic vessels from 4T1 mice treated with docetaxel appeared larger compared to control IgG-treated mice, and this size increase was mitigated by anti-VEGFR3 therapy (Fig. 4). Quantification of the size of vessels revealed a significant increase in both lymphatic vessel perimeter and area (Fig. 5a, b) in docetaxel-treated tumor-draining lymphatics. This effect was significantly attenuated by adjuvant VEGFR3 inhibition, reducing the vessel size below that of the control IgG-treated vessels. Docetaxel also significantly increased lymphatic vessel number in the tumor stroma, an indicator of lymphangiogenesis, which was significantly attenuated by anti-VEGFR3 therapy (Fig. 5c). The differences in lymphatic vessel size (R^2^ = 0.0057, n.s.) and density (R^2^ = 0.20327, n.s.) were not correlated with tumor size, suggesting that these effects are not an artifact of the differences in tumor growth across treatment groups. Interestingly, these changes to lymphatics with docetaxel were tumor-dependent and did not occur within tumor-naïve contralateral fat pads (Fig. 5a-c). Quantification of LEC number in tumor-bearing mammary fat pads by flow cytometry (Fig. 5d) revealed expansion of the LEC population (gp38+/CD31+/CD45-) [22] with docetaxel treatment, which was significantly mitigated by VEGFR3 blocking. The docetaxel-induced increases in lymphatic vessel size and number led us to question whether this vessel dilation resulted in functional changes such as fluid drainage, as lymphatic dilation has been shown to enhance fluid drainage capacity [14, 26]. Docetaxel treatment led to a 50% increase in fluid drainage from the tumor to the tumor-draining axillary lymph node and VEGFR3 inhibition resulted in significantly decreased drainage, as determined by total Evans blue dye in axillary nodes following intratumoral injection (Fig. 5e). While we have shown that a single dose of docetaxel is sufficient to cause widespread changes to the lymphatic vasculature, we were curious to see effects after multiple rounds of docetaxel treatment to enhance the clinical relevance of these findings, as patients would undergo chronic treatment in the clinic. Interestingly, we observed that chronic docetaxel treatment exacerbated the lymphangiogenic effect of docetaxel (Fig. 5f), significantly increasing lymphatic vessel density in a dose-dependent manner from 0.5 vessels/mm^2^ stroma with 1 dose to almost 1.25 vessels/mm^2^ after 3 doses. Together, we demonstrate that docetaxel alters lymphatics in ways widely regarded as hallmarks of lymphatic activation [13], including dilation and lymphangiogenesis, and these changes can be mitigated by anti-VEGFR3 adjuvant therapy.

### Docetaxel treatment increases expression of pro-lymphangiogenic cytokines in the tumor microenvironment

Studies using both human samples and animal models have shown that peritumoral lymphatic vessels undergo remodeling events, such as enlargement and lymphangiogenesis, as a result of lymphatic activation [13]. A variety of secreted factors produced by both cancer and stromal cells can induce these phenomena [13], either by inducing proliferation of LECs to promote new vessel formation or sprouting, causing morphological changes to LEC shape to expand the size of the vessel [14], or both. Given the dramatic changes to lymphatic vessel morphology and density that we observed after docetaxel treatment in vivo, we hypothesized that docetaxel could be causing an increase in pro-lymphangiogenic factors in the breast tumor microenvironment that may mediate these effects.

**Fig. 6:**
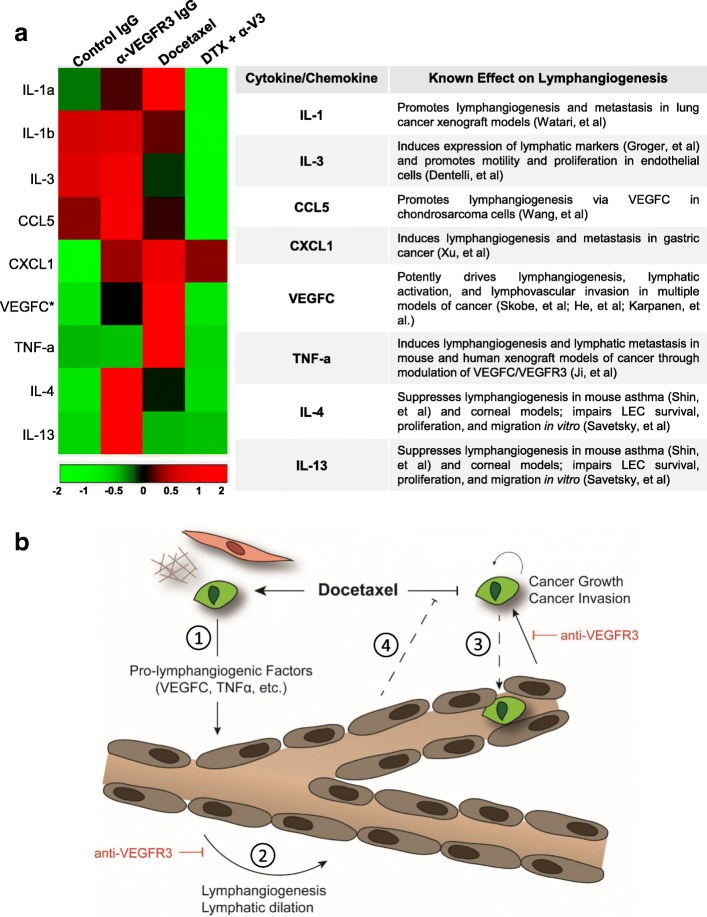
Docetaxel treatment increases expression of pro-lymphangiogenic cytokines in 4T1 tumors. **a** Heat map representation of expression of chemokines associated with lymphangiogenesis (left) and corresponding known roles in cancer and lymphangiogenesis (right). Results obtained by flow cytometry analysis of 4T1 tumors treated as outlined in Fig. 2 (*n* = 4/group). Log-transformed data displayed as fold change and heat map generated using MatLab software. (*) indicates analysis by ELISA. Docetaxel abbreviated as DTX and anti-VEGFR3 therapy abbreviated as α-V3 in figure. **b** Proposed mechanism by which docetaxel results in lymphatic activation and the resulting effect on cancer cell response to therapy. (1) Docetaxel induces production of pro-lymphangiogenic factors in the breast tumor microenvironment. (2) Docetaxel-induced lymphangiogenic factors like VEGFC and TNFa result in VEGFR3-dependent enlargement of lymphatics (brown) and lymphangiogenesis. (3) Docetaxel-activated lymphatics promote VEGFR3-mediated tumor cell (green) invasion and (4) reduce docetaxel efficacy

We therefore treated 4T1 mice with anti-VEGFR3 or IgG control antibody, followed by a single dose of systemic docetaxel or vehicle control, and examined expression of a variety of chemokines and cytokines 24 h post-treatment in the tumor-bearing mammary fat pads by multiplex analysis (Fig. 6a, Additional file 2: Table S2). We identified a host of factors associated with lymphangiogenic correlates were affected by therapy. Most notably, docetaxel treatment of 4T1 tumors resulted in a nearly two-fold increase in both VEGFC and TNF-α (*p* < 0.05, IgG v. Docetaxel), both of which are potent drivers of VEGFR3-dependent lymphangiogenesis and lymphatic activation [27, 11]. Addition of anti-VEGFR3 therapy effectively reduced VEGFC and TNF-α (*p* < 0.05, IgG v. Combo) back to baseline levels. In addition to VEGFC and TNF-α, we found a number of other chemokines and cytokines associated with lymphangiogenesis that were also altered in response to therapy. Docetaxel increased expression of IL-1α (*p* < 0.05, IgG v. Docetaxel), which has been implicated in promoting inflammatory lymphangiogenesis and lymph node metastasis in an aggressive lung cancer model [28], and CXCL1 (*p* < 0.05, IgG v. Docetaxel), a chemokine secreted by LECs found to induce lymphangiogenesis and metastasis in gastric cancer [29], in 4T1 tumors. Interestingly, anti-VEGFR3 therapy resulted in increases to anti-lymphangiogenic Th2 cytokines IL-4 (*p* < 0.001, IgG v. anti-VEGFR3) and IL-13 (*p* < 0.1, IgG v. anti-VEGFR3), both of which have been found to inhibit LEC proliferation and activation and suppress lymphangiogenesis in both corneal and asthma murine models [30]. Anti-VEGFR3 treatment also yielded heightened expression of CCL5 (*p* < 0.0001, IgG v. anti-VEGFR3), which may account for the modest increase in tumor growth observed in that treatment cohort as CCL5 has been shown to accelerate tumor growth in some mouse models [31–33].

In addition, when 4T1 tumors were treated with a combination of anti-VEGFR3 therapy and docetaxel chemotherapy, we observed a synergistic reduction to other pro-lymphangiogenic factors such as IL-1α (*p* < 0.1, anti-VEGFR3 v. Combo; Docetaxel v. Combo), IL-1β (*p* < 0.001, anti-VEGFR3 v. Combo; *p* < 0.1, Docetaxel v. Combo), IL-3 (*p* < 0.001, anti-VEGFR3 v. Combo; *p* < 0.1, Docetaxel v. Combo), and CCL5 (*p* < 0.0001, anti-VEGFR3 v. Combo; *p* < 0.05, Docetaxel v. Combo), a chemokine shown promote lymphangiogenesis through upregulation of VEGFC expression by chondrosarcoma cells [34]. Together, these findings show docetaxel increases expression of multiple pro-lymphangiogenic factors, including VEGFC and TNF-α, in the 4T1 tumor microenvironment, and these changes can be mitigated by the addition of anti-VEGFR3 therapy.

## Discussion

We have shown that docetaxel, a common chemotherapy against breast cancer, can induce lymphangiogenesis in tumor-associated lymphatics and that these lymphatic alterations can in turn increase tumor invasion and reduce response to docetaxel. We propose that this occurs in part via a complex intercellular mechanism that includes tumor cells, lymphatic endothelial cells, and chemotherapy (Fig. 6b).

### Docetaxel upregulates pro-lymphangiogenic factors in the breast tumor microenvironment (1)

A host of factors associated with lymphangiogenesis and tumor progression were upregulated following treatment with docetaxel, including VEGFC, TNF-α, IL1, among others. Interestingly, the majority of the pro-inflammatory cytokines and chemokines upregulated by docetaxel in our study are downstream of TLR4-activated NF-κB signaling [35]. TLR4 is expressed in a number of tumor cell lines, including 4T1 [36] and MDA-MB-231 [37, 38], as well as LECs [39]. TLR4 can be directly activated by taxanes [40–43, 35]. Our data show that when VEGFR3 signaling is inhibited during docetaxel treatment, there is a synergistic reduction in other pro-lymphangiogenic factors, such as IL-1α, IL-1β, IL3, and CCL5. Activation of TLR4 by paclitaxel has been shown to upregulate VEGFR3 [44], which may account for some of the VEGFR3-dependence of these taxane-induced phenomena.

The induction of these cytokines by docetaxel can have profound effects on lymphatics. For example, we observed an upregulation of VEGFC in 4T1 tumor lysates. Similar increases in overall VEGFC in breast tumors in vivo were reported by Alishekevitz, et al. with paclitaxel treatment [20]. The VEGFC-VEGFR3 signaling axis is one of the quintessential drivers of tumor-associated lymphangiogenesis [13]. Since other cancers receive taxane-based treatment, including non-small cell lung cancer [45], metastatic castration-resistant prostate cancer [46], and gastric cancer [47], our findings here may extend to other tissues. As with TNBC, in these cancers, increased expression of VEGFC correlates with poorer prognosis. VEGFC also triggers chemotaxis in subsets of lymphatic endothelial progenitor cells and macrophages [48]. In fact, VEGFR3+ pro-lymphangiogenic macrophages were shown to be recruited to paclitaxel-treated tumors and may be responsible, in part, for the increased lymphangiogenesis seen in our models [20]. These unique cells both respond to pro-lymphangiogenic signals and encourage lymphangiogenesis in tissues, as discussed by Corliss, et al. [48] Other factors identified in our cytokine analysis, such as TNF-α, have been associated with increased lymphangiogenesis, increased immune cell recruitment, and stromal activation [49]. A recent study showed a novel mechanism by which the TNF-α:TNFR1 and VEGFC:VEGFR3 signaling pathways coordinate to promote lymphangiogenesis and lymphatic metastasis [27, 50]. Together, these pro-lymphangiogenic factors can synergize to create a tumor microenvironment that encourages tumor progression and recurrence [49, 51].

### Docetaxel treatment of tumor results in lymphangiogenesis and enlargement of lymphatics (2)

In vivo, docetaxel treatment resulted in increased LEC numbers, vessel density, and vessel size, indicative of lymphangiogenesis in a VEGFR3-dependent manner. Studies using both human samples and animal models have shown that peritumoral lymphatic vessels undergo morphological changes [13]. Enlargement of vessels can occur by inducing proliferation of LECs or causing changes to LEC shape that expand the size of the vessel as a whole [14]. Tumor-induced increases in the diameter of collecting lymphatics is associated with enhanced passage of clusters of tumor cells to the tumor-draining lymph nodes [14]. This greater surface area of potential contact between tumor cells and lymphatic vessels can also increase invasive entry into the vessels [13] and have broad-reaching effects on lymphatic-immune interactions [52]. Increased proliferation and dilation of lymphatic vessels have been shown to enhance fluid drainage capacity [14, 26]. Docetaxel treatment coincided with an increase in lymphatic drainage to axillary nodes in mice, in line with similar changes seen with standard VEGFC-induced lymphangiogenesis [53]. Increased drainage has been shown to correlate with increased interstitial flow in the peritumoral tissue, and this increased fluid flow has been linked to increased tumor cell invasion [54–56], fibroblast activation [57], and altered immunological function [58]. In line with these findings, we detected enhanced tumor cell metastasis in lymph nodes of 4T1 mice treated with docetaxel. We did not observe increased metastasis to lungs, though others have observed increases in lung metastasis with paclitaxel treatment when tumors were allowed to grow beyond our humane endpoints [20]. However, we believe that lymphatic metastasis to the lymph node is a more direct indicator of the counter-therapeutic effects that docetaxel exerts on the lymphatic vasculature. These data indicate that docetaxel may be priming lymphatics in ways that could contribute to increased metastatic spread of tumor cells.

Lymphatic alterations were unique to the tumor-bearing fat pads of mice and were not seen with docetaxel treatment in naïve fat pads, indicating the need for tumor cells to elicit docetaxel-dependent lymphangiogenesis. VEGFC, the primary mediator of lymphangiogenesis in cancers, has been overexpressed in a number of models leading to increased lymphatics [11, 19]. In these models, lymphangiogenesis occurs intratumorally, which is not representative of the relative lack of intratumoral lymphatics in breast cancer patients [59–61]. Here, we see increased peritumoral lymphatics, more in line with patient data of lymphatic localization, and it is these lymphatics that respond to treatment. We saw increased VEGFC secretion by cancer cells in response to docetaxel treatment. Others have suggested that VEGFC-expressing pro-lymphangiogenic macrophages are responsible for mediating lymphangiogenesis in breast cancer, showing that paclitaxel-induced lymphangiogenesis could be ameliorated by depleting this population of cells [20]. However, tumors in these mice were analyzed for lymphatics at a much later time point, when tumors are large and stroma is sparse, compared to our data where stroma is still a major component of the fat pad. Thus, we find that docetaxel increases lymphangiogenesis in tumor-bearing fat pads and this is mediated in part by VEGFC, but also by a host of other pro-lymphangiogenic cytokines likely induced by TLR4 pathway activation by taxanes as discussed earlier.

### Docetaxel-treated tumor-educated lymphatics promote cancer cell invasion in a VEGFR3-dependent manner (3)

Docetaxel treatment in both MDA-MB-231 and HCC38 cell lines increased tumor cell invasion only when LECs were present in our in vitro system, and this docetaxel-stimulated invasion was attenuated by VEGFR3 inhibition. It has been well established that LECs stimulated by VEGFC undergo a number of activating cellular changes that can be hijacked by cancer cells to facilitate invasion [62]. Accordingly, we found that docetaxel enhanced lymph node metastasis of breast cancer cells in vivo, closely paralleling our human in vitro findings showing increased cancer cell invasion toward lymphatics after docetaxel treatment.

Interestingly, in vitro, we selected three cell lines representing disparate molecular subtypes of TNBC [4] and these lines responded differently towards LECs. MDA-MB-231, a mesenchymal stem-like line, increased invasion in the presence of LECs. This subtype is characterized by increased expression of motility-supporting genes, and thus alterations to motility are not surprising in response to stimuli [5]. HCC38, a basal-like 2 cell line, required docetaxel treatment to invade towards LECs. This subtype is particularly enriched for growth factor signaling pathways [5]. HCC1806, a basal-like 1 subtype, was never LEC responsive. Basal-like 1 cells are highly proliferative but show little propensity for invasion and metastasis as evidenced by their better overall relapse-free survival and reduced hazard of distant-metastasis compared to mesenchymal subtypes [5]. Interestingly, the HCC1806 cell line does not express TLR4 [63]. It is possible that the behaviors we are observing in response to lymphatics are representative of the inherent behavior of these subtypes. However, it is very likely that the lymphangiogenic changes in cytokine and chemokine signaling caused by docetaxel is dependent on TLR4 pathway activation and without it, the cancer cell does not produce the necessary LEC-activating signals required to promote their invasion.

### Lymphatics reduce efficacy of docetaxel [4]

The significant decrease in cancer cell death after treating with docetaxel in both MDA-MB-231 and HCC38 cell lines was dependent on the presence and number of LECs, but not directly on VEGFC/VEGFR3 signaling*.* In vivo*,* VEGFR3 blockade had a similar anti-tumor effect in combination with docetaxel, but this treatment directly reduces total LEC numbers in the tumor. Therefore, the LEC-mediated reduction in chemotherapeutic death may be dependent on the number of LECs present which is reduced in vivo by VEGFR3 blockade but not in vitro, where LEC numbers remain constant due to contact inhibition within the in vitro system. The contribution of stromal cells to reduced therapeutic response has been shown with myeloid cells, fibroblasts, and blood endothelial cells, all of which, when reduced in the tumor microenvironment, similarly enable better response to chemotherapy [20, 64–66]. Tumor-educated LECs were recently shown to secrete EGF, which contributed to increased cancer cell proliferation and tumor growth in vivo, as well as PDGF-BB, which enhanced pericyte infiltration and angiogenesis [67]. EGF may also be involved in reducing docetaxel efficacy in cancer cells; however, a number of other molecules including IL-6 [68, 69], TGFβ [70], CXCL12 [64], CCL21 [71], VEGF [72], among others secreted by activated lymphatics are known contributors to drug resistance and these interactions may be tumor cell specific. Regardless, our data show that LECs can desensitize cancer cells to taxane-induced death in vitro and reduction of LEC numbers in vivo results in better response to therapy. Recent studies have begun to illustrate a role for LECs in cancer growth [67] and the potent reduction in chemotherapeutic efficacy in the presence of LECs that we have demonstrated complements and builds upon these findings to underscore the importance of lymphatics in cancer cell response to therapy.

#### Clinical implications

Normalization of vasculature in cancer has been an area of therapeutic interest for many years [73]. Targeting blood vessel angiogenesis with VEGFR2-targeted therapy, such as bevacizumab, has been a focus of the majority of this research; however, this therapy does not reduce metastasis in breast cancer [74]. The role of activated tumor-associated lymphatic vessels in the metastatic spread of cancer has been well-established in many types of cancer [26]. While LEC survival during development is dependent on VEGFR3, mature lymphatic vasculature found in adults is usually quiescent. [62]. Thus, in the context of cancer, LEC signaling through the VEGFR3 pathway is mostly restricted to tumor-associated lymphatic vessels, making VEGFR3 an ideal candidate for therapeutic intervention. However, specific anti-VEGFR3 therapy has not been shown in preclinical studies to have anti-tumor effects alone [12–17] with studies focusing on the anti-metastatic benefits of this therapy. Thus, anti-VEGFR3 is unsuitable as a single agent therapy for patients in need of reduction in primary tumor burden. Based on our data here, combined with other reports showing combinatorial benefit [20], anti-VEGFR3 may be an ideal supplement to current standard of care chemotherapy. Our study not only suggests that there is synergistic anti-cancer benefits when docetaxel and anti-VEGFR3 therapy are administered together, but also underscores the benefit of anti-VEGFR3 to counteract docetaxel-induced lymphatic changes which could contribute to longer term treatment-associated issues and recurrence.

## Conclusions

Though most breast tumors will be treated with chemotherapy during clinical care, the effects of common chemotherapeutics like docetaxel on the cancer stroma remain largely unknown; consequences of chemotherapy on lymphatics are particularly understudied. Together, our findings illustrate a novel mechanism by which tumor-associated lymphatics become activated by docetaxel, which may reduce docetaxel efficacy on cancer growth and promote metastasis. These data may prove highly relevant to the design of clinical cancer care regimens that include anti-VEGFR3 in the future.

## Additional files

Additional file 1: **Figure S1.** LECs increase VEGFR3-dependent invasion of human breast cancer cells after treatment with docetaxel in human 3D in vitro model of breast tumor microenvironment. Representative images of invaded tumor cells in 3D in vitro system after treatment with docetaxel and VEGFR3 inhibitor MAZ51. **Figure S2.** Blockade of VEGFR3 in combination with docetaxel reduces primary tumor growth and lung metastasis. Representative images and quantification of lung metastasis in treated 4T1 mice. Representative bioluminescent images of 4T1 tumors in treated mice. **Table S1.** LECs increases EC50 of docetaxel in three human breast cancer cell lines. Cell death EC50 of docetaxel in three human breast cancer cell lines with or without LECs in 3D in vitro system. **Figure S3.** LEC-mediated reduction in docetaxel-induced cytotoxicity is independent of VEGFR3. Cell death of three human breast cancer cell lines with or without LECs in 3D in vitro system with VEGFR3 inhibition. (PDF 4471 kb)

Additional file 2: **Table S2.** Luminex Data. Luminex data used to generate heat map in Fig. 6. (XLSX 11 kb)

## Abbreviations

HMF: Human mammary fibroblasts
LECs: Lymphatic endothelial cells
TNBC: Triple-negative breast cancer
VEGFC: Vascular endothelial growth factor C
VEGFR3: Vascular endothelial growth factor receptor 3
