# Supplementary material for: Docetaxel facilitates lymphatic-tumor crosstalk to promote lymphangiogenesis and cancer progression
Source: BMC Cancer. 2018 Jul 6;18:718. doi: 10.1186/s12885-018-4619-8 (PMC6034223; doi:10.1186/s12885-018-4619-8)
Supplement: Supplementary file 1 — Figure S1. LECs increase VEGFR3-dependent invasion of human breast cancer cells after treatment with docetaxel in human 3D in vitro model of breast tumor microenvironment. Representative images of invaded tumor cells in 3D in vitro system after treatment with docetaxel and VEGFR3 inhibitor MAZ51. Figure S2. Blockade of VEGFR3 in combination with docetaxel reduces primary tumor growth and lung metastasis. Representative images and quantification of lung metastasis in treated 4T1 mice. Representative bioluminescent images of 4T1 tumors in treated mice. Table S1. LECs increases EC50 of docetaxel in three human breast cancer cell lines. Cell death EC50 of docetaxel in three human breast cancer cell lines with or without LECs in 3D in vitro system. Figure S3. LEC-mediated reduction in docetaxel-induced cytotoxicity is independent of VEGFR3. Cell death of three human breast cancer cell lines with or without LECs in 3D in vitro system with VEGFR3 inhibition. (PDF 4471 kb) [file 12885_2018_4619_MOESM1_ESM.pdf]

Figure S1 | LECs increase VEGFR3-dependent invasion of human breast cancer cells after treatment with docetaxel in human 3D *in vitro* model of breast tumor microenvironment.

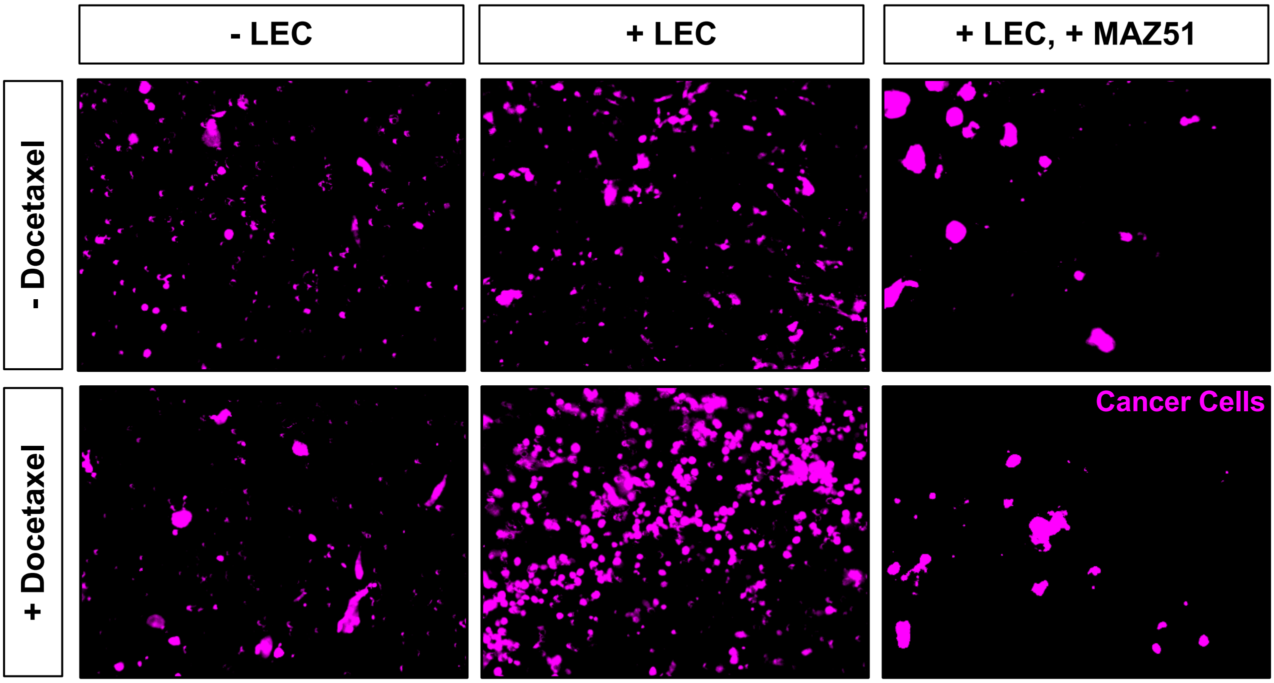

Figure S1 | LECs increase invasion of human breast cancer cells after treatment with docetaxel in human 3D *in vitro* co-culture system. Representative images of cancer cell invasion of pre-labeled MDA-MB-231 cells in our 3D microenvironment system following treatment with and without docetaxel (0.1 μM) with and without MAZ51 (1 μM) in the presence or absence of LECs, as described in Fig. 1.

Figure S2 | Blockade of VEGFR3 in combination with docetaxel reduces primary tumor growth and lung metastasis in 4T1.

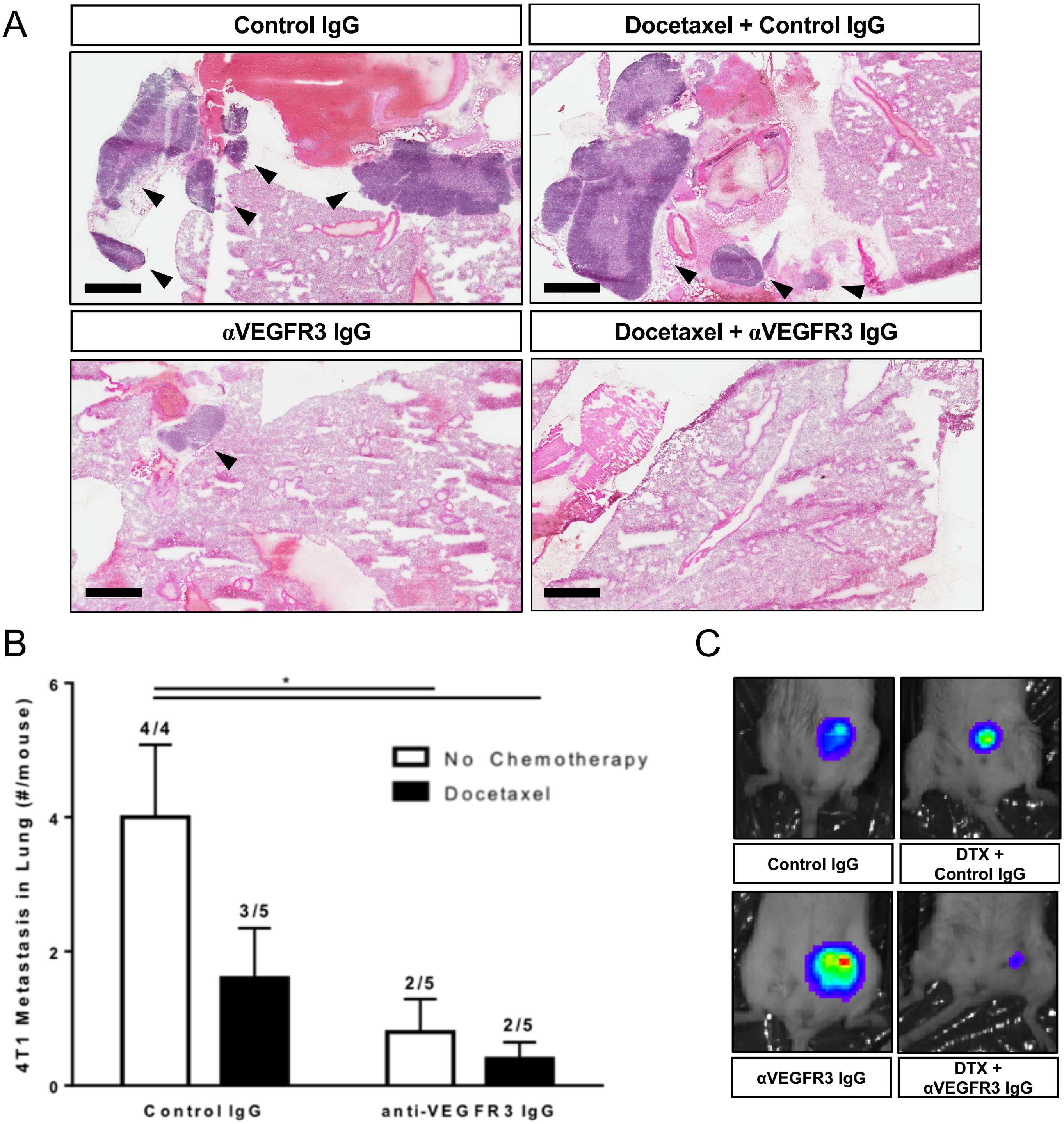

Figure S2 | Blockade of VEGFR3 in combination with docetaxel reduces primary tumor growth and lung metastasis in 4T1. (A) Representative H&E images of lung metastases from 4T1 mice treated with docetaxel and/or anti-VEGFR3. Metastatic lesions are noted by black arrowheads. (n=5/cohort) **(B)** Quantification of lung metastasis. Columns represent number of metastatic foci per mouse. Numbers above columns represent number of mice in each cohort that developed any lung metastasis *p< 0.05. (n=5). **(C)** Representative bioluminescence imaging of 4T1-luciferase tumors in mammary fat pads of mice treated with docetaxel (or vehicle) and anti-VEGFR3 antibody (or control IgG) as previously described in Figure 2.

Table S1 | LECs increase EC50 of docetaxel in three human breast cancer cell lines**.**

Cell Death EC50 of Docetaxel (µM)					
HCC 38		MDA-MB-231		HCC 1806	
**- LEC**	**+ LEC**	**- LEC**	**+ LEC**	**- LEC**	**+ LEC**
0.052	101.2	0.3	81.2	513.7	657.0

Table S1 | LECs increase EC50 of docetaxel in three human breast cancer cell lines. EC50 calculated for cancer cell death as quantified by flow cytometry following docetaxel treatment in 3D *in vitro* system for HCC38, MDAMB231, and HCC1806 human breast cancer cell lines with or without LECs present in the system as described in Fig. 1.

Figure S3 | LEC-mediated reduction in docetaxel-induced cytotoxicity is independent of VEGFR3.

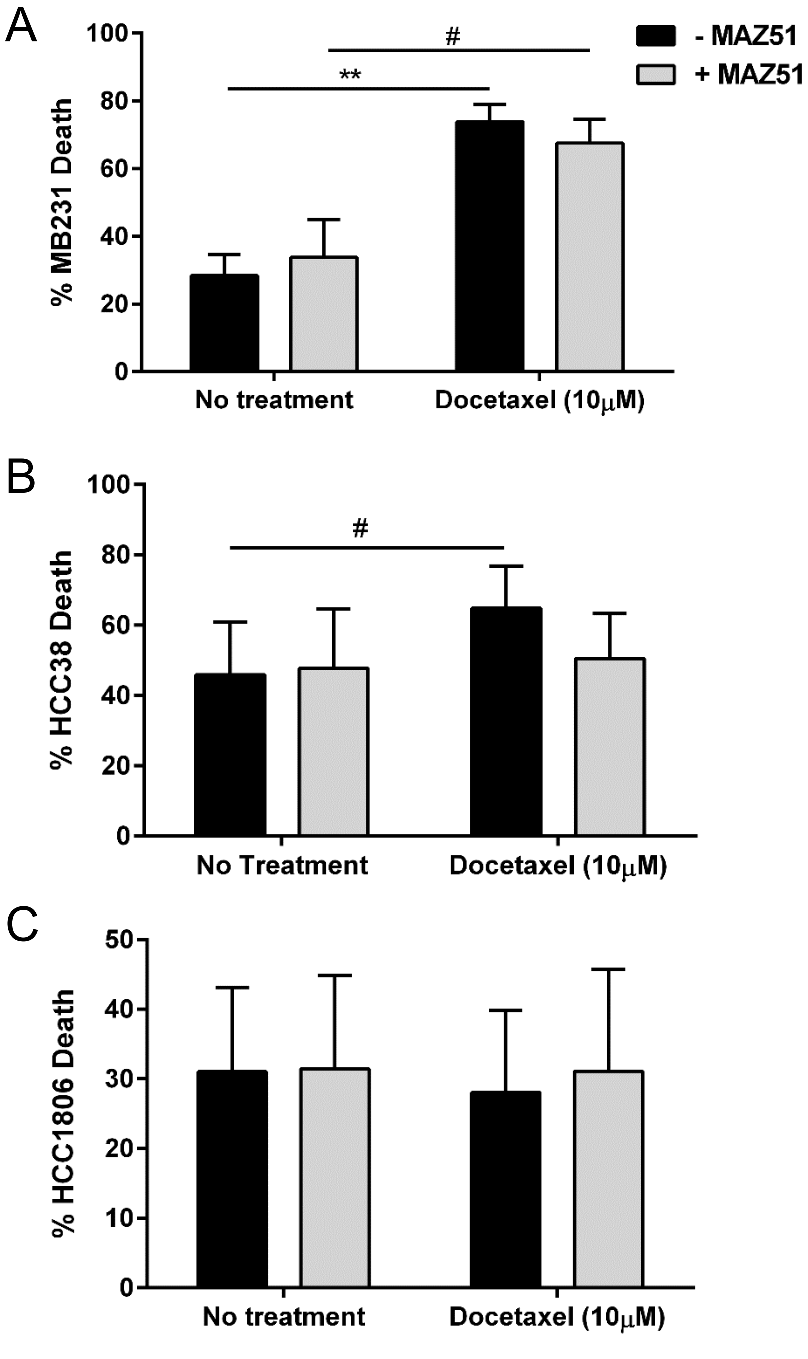

Figure S3 | LEC-mediated reduction in docetaxel-induced cytotoxicity is independent of VEGFR3. (A) Cancer cell death of MDA-MB-231 TNBC cells in 3D microenvironment system with and without docetaxel treatment (10 μM) in the presence of LECs treated with or without VEGFR3 inhibitor MAZ51. **(B)** Cancer cell death of HCC 38 TNBC cells in 3D microenvironment system with and without docetaxel treatment (10 μM) in the presence of LECs treated with or without VEGFR3 inhibitor MAZ51. **(C)** Cancer cell death of HCC 1806 TNBC cells in 3D microenvironment system with and without docetaxel treatment (10 μM) in the presence of LECs treated with or without VEGFR3 inhibitor MAZ51. Results are indicated as % dead cancer cells as assessed by flow cytometry. #p<0.1, **p<0.01
